# Evaluation of prodynorphin gene polymorphisms and their association with heroin addiction in a sample of the southeast Iranian population

**DOI:** 10.22099/mbrc.2017.27182.1294

**Published:** 2018-03

**Authors:** Mohammad Hashemi, Mansour Shakiba, Sara Sanaei, Ghazaleh Shahkar, Maryam Rezaei, Azizolla Mojahed, Gholamreza Bahari

**Affiliations:** 1 Cellular and Molecular Research Center, Zahedan University of Medical Sciences, Zahedan, Iran; 2 Department of Clinical Biochemistry, School of Medicine, Zahedan University of Medical Sciences, Zahedan, Iran; 3 Department of Psychiatry, School of Medicine, Zahedan University of Medical Sciences, Zahedan, Iran; 4 Department of Clinical Psychology, School of Medicine, Zahedan University of Medical Sciences, Zahedan, Iran

**Keywords:** Prodynorphin, Addiction, Heroin, Polymorphism, VNTR

## Abstract

Genetic factors are supposed to account for about 30-50% of the predisposition to cocaine and heroin addiction. This study aims at investigating the effect of rs2281285, rs2235749, rs910080 and 68bp VNTR polymorphisms of prodynorphin (*PDYN*) gene on heroin dependence risk in a sample of the southeast Iranian population. This case-control study was done on 216 heroin dependence subjects and 219 healthy subjects. Genomic DNA was extracted from peripheral blood cells using salting out method. Genotyping of *PDYN* polymorphisms were performed using polymerase chain reaction (PCR) or PCR-RFLP method. The findings showed that *PDYN* rs910080 T>C variant significantly increased the risk of heroin dependence (OR=7.91, 95%CI=3.36-18.61, P<0.0001, CC vs TT; OR=7.53, 95%CI=3.30-17.16, P<0.0001, CC vs TT+TC; OR=1.75, 95%CI=1.33-2.32, p<0.0001, C vs T). The rs2235749 C>T, rs2281285 A>G and 68bp VNTR variants of *PDYN* gene were not associated with heroin dependence. Altogether, our results provide an association between rs910080 polymorphism of *PDYN* gene and risk of heroin dependence in a sample of the southeast Iranian population.

## INTRODUCTION

It has been suggested that both genetic and environmental factors contribution to individual differences in vulnerability to drug addictions [1]. Drug addiction is described as a chronic disease characterized by compulsive drug seeking, drug abuse, physical dependence and tolerance [2]. Twin studies have proposed that genetic factors account for approximately 30-60% of the overall variance in the risk of developing drug addiction [3-5].

The *PDYN* gene mapped on short arm of chromosome 20 (20p13) [6]. PDYN is the precursor of the dynorphin related peptides. The PDYN plays key role in some complex traits including drug abuse [7]. DYNs, which are posttranslational products of *PDYN* gene, bind to opiate receptors, but bind with high affinity to kappa-opioid receptor (KOR) [8, 9]. DYN inhibits the release of dopamine and is consequently supposed to play an important role in the negative feedback regulation of dopamine [10-12]. KOR and DYNs are enriched in brain circuits that control mood, motivation, and stimulus-response (habit) formation and have been involved in drug-seeking behavior [13]. 

*PDYN* gen is polymorphic and the alterations of *PDYN* expression may be affected by functional polymorphisms [14]. Several studies have examined the relationship between *PDYN* polymorphisms and opioid addiction, but the findings were inconsistent [15-20]. Some investigations statement that the variant of *PDYN* significantly increased the risk of opioid and cocaine addiction [15-17, 20]; though, other studies proposed that the variant decreased the risk of addiction [18, 19]. Therefore, the present study aimed to examine the possible association between rs2235749 and rs910080 variants in the 3`-untranslated regions (3`UTR), the rs2281285 variant in the second intron, and the 68-bp VNTR polymorphism in the promoter region of *PDYN* gene with the risk of heroin addiction in a sample of the southeast Iranian population.

## MATERIALS AND METHODS

**Patients:** This case-control study was done in Zahedan (southeast Iran) on 216 heroin addicts (39 females and 177 males, ages 38.4±12.1) who referred to Baharan Hospital (Psychiatric hospital of Zahedan University of Medical Sciences) for methadone maintenance therapy and 219 controls (44 females and 175 males, ages 36.2±11.0) who declared that they did not suffer substance abuse. The local Ethics Committee of the Zahedan University of Medical Sciences approved the project, and written informed consent was taken from all individuals. Two milliliter of venous blood was drawn from each participant and genomic DNA was extracted by using salting out method. 

**Genotyping:** Genotyping of the rs2281285, rs2235749, rs910080 was performed by polymerase chain reaction-restriction fragments length polymorphism (PCR-RFLP). The 68bp VNTR polymorphism was genotyped by PCR method. The primer sequences for genotyping of *PDYN* variants, restriction enzymes and length of the fragments are summarized in Table 1. The 20 µl reaction mixture contained 1 µl of genomic DNA (~100 ng/µl), 1 µl of primers (10 µM), 10 µl of 2X Prime Taq Premix (Genet Bio, Korea) and 7 µl ddH2O. Thermocycler conditions were as follows: 95˚C for 5 min, 30 cycles of 95˚C for 30 s, annealing temperature (Table 1) for 30 s, and 72˚C for 30 s and a final extension step of 72˚C for 10 min. Ten microliter of PCR product were digested by appropriate restriction enzyme (Table 1) and the fragments were resolved on 2.5% agarose gel electrophoresis. Alleles with 3 or 4 repeats have been designated as high (H) expression alleles and those with 1 or 2 as low (L) expression alleles [27].

**Statistical analysis:** Independent sample t-test or χ2 test was used to compare the variable between the groups according to the data. Odds ratio (OR) with 95% confidence intervals (CIs) was calculated from logistic regression analyses to find out the impact of the polymorphisms on heroin addiction. Statistical calculations were achieved using SPSS 22 software.The level of significance was set as p<0.05. 

**Table 1:** The primers used for detection of *PDYN* polymorphisms using PCR-RFLP or PCR methods

**polymorphisms**	**Primer sequence (5`->3`)**	**Annealing (˚C)**	**Restriction enzyme**	**Fragments (bp)**
rs910080 A>G	F: CAATGCCCAGTGCGTATGT R: CTTTGGAGACGATGCTTTAGGT	65	Bsp1286I	T allele, 497; C allele, 300+197
rs2235749 A>G	F: TGGAAACCAAGACATCAGG R: TCATTGTTCAGAAAAGCACC	62	NdeI	C allele, 571; T allele, 365+206
rs2281285 A>G	F: GCTCAGATTTTCACTGTTCCGA R: AGCCAACATTCATGGGCTGA	60	HPY8I	A allele, 423; G allele, 240+183
68-bp VNTR	F: ATCCAAGGTCTCTCCGATGGT R: CACCAGGCGGTTAGGTAGA	68	-	Alleles containing 1, 2, 3, or 4 repeats, produced fragments of 350, 418, 486, and 554

## Results

In total 216 heroin addicts (39 females and 177 males, ages 38.4±12.1) who referred to Baharan Hospital (Psychiatric hospital of Zahedan University of Medical Sciences) for methadone maintenance therapy and 219 controls (44 females and 175 males, ages 36.2±11.0) who declared that they did not suffer substance abuse were included in the study. No significant difference was found between the groups regarding age (p=0.057) and sex (p=0.627).

Table 2 shows the genotypic frequencies of *PDYN* polymorphisms in heroin addiction and healthy subjects. The findings proposed that *PDYN* rs910080 variant significantly increased the risk of heroin dependence (CC *vs* TT: OR=7.91, 95%CI=3.36-18.61, P<0.0001). While, the results did not show any significant association between rs2235749 C>T, rs2281285 A>G and 68bp VNTR variants of *PDYN* gene and heroin dependence. The genotypic frequency of the rs910080 and rs2235749 polymorphisms in controls were not consistent with Hardy-Weinberg eqilibrium (P<0.05).

**Table 2:** Genotypic frequencies of the *PDYN* polymorphisms in heroin dependent persons and healthy control subjects

**Polymorphisms**	**Cases n (%)**	**Controls n (%)**	**OR (95% CI)**	**p**
**rs910080 **				
TT	73 (33.8)	94 (42.9)	1.00	-
TC	100 (46.3)	118 (53.9)	1.09 (0.73-1.64)	0.681
CC	43 (19.9)	7 (3.2)	7.91 (3.36-18.61)	<0.0001
				
**rs2235749**				
CC	68 (31.5)	65 (29.7)	1.00	-
CT	131 (60.6)	145 (66.2)	0.86 (0.57 -1.31)	0.527
TT	17 (7.9)	9 (4.1)	1.81 (0.75-4.34)	0.203
				
**rs2281285**				
AA	129 (59.7)	147 (67.1)	1.00	-
AG	82 (38.0)	68 (31.1)	1.37 (0.92-4.05)	0.129
GG	5 (2.3)	4 (1.8)	1.42 (0.37-5.42)	0.739
				
**68bp VNTR**				
3/3	79 (36.6)	85 (38.3)	1.00	-
2/2	26 (12.0)	27 (12.3)	1.04 (0.56-1.93)	0.911
2/3	95 (43.5)	95 (42.9)	1.08 (0.71-1.64)	0.750
3/4	8 (3.7)	4 (1.8)	2.15 (0.62-7.43)	0.246
2/4	8 (3.7)	8 (3.7)	1.07 (0.38-3.01)	0.889

## DISCUSSION

Alterations in the *PDYN* gene expression might be influenced by genetic polymorphisms and epigenetic mechanisms. Consequently, genetic variations or epigenetic changes of the *PDYN* may be a risk factor drug abuse susceptibility [21, 22]. Some studies evaluated the impact of *PDYN* polymorphisms on heroin dependence but, the available findings remained inconsistent [15-19]. In the present study, we inspected the possible association between *PDYN* rs910080, rs2281285, rs2235749, and 68bp VNTR polymorphisms and heroin dependence in a sample of southeast Iranian population. The findings suggest that *PDYN* rs910080 T>C variant significantly increased the risk of heroin dependence. No significant association was found between rs2281285, rs2235749, and 68bp VNTR polymorphisms and heroin dependence.

Growing evidence propose that dynorphin/kappa-opioid receptor system plays a significant role in alcohol and drug dependence [21, 23-25]. Saify et al [20] investigated the impact of VNTR polymorphism on heroin addiction in Shiraz, southern Iran. In contrast to our findings, they found that HL genotype and L allele significantly increased the risk of heroin addiction. Stratification by sex revealed that VNTR variant was associated with the risk of heroin addiction only in male [20]. In another study, Saify et al [26] have found no significant association between the VNTR polymorphism in the promoter region of the *PDYN* gene and the risk of methamphetamine dependence. Zimprich et al [27] have found no significant association between 68-bp VNTR polymorphism located in the *PDYN* gene promoter region and heroin addiction. One to four repeats of a 68-bp element comprising one binding site per repeat for the transcription factor AP-1 (c-Fos/c-Jun). It has been shown that alleles 3 or 4 repeats are associated with higher expression of dynorphin peptides and higher degrees of dopamine inhibition than that of alleles with 1 or 2 repeats [27]. 

It has been proposed that the long alleles (three or four repeats) of *PDYN* VNTR may be a risk factor for developing cocaine/alcohol codependence in the African American population [28, 29]. The 68-bp VNTR polymorphism of *PDYN* is a functional variant and influence gene expression [27]. Individuals carrying 3 or 4 copies of VNTR *PDYN* promoter exhibit higher PDYN expression than those with only 1 or 2 copies [18, 27]. Following studies revealed that the impact of 68-bp VNTR polymorphism on expression of PDYN is cell type dependent [30]. 

D'Addario et al [31] have shown that *PDYN* rs2235751 variant was associated with alcoholism and the presence of the minor allele G was associated with reduced *PDYN* promoter DNA methylation in females and younger subjects. 

Recently, Egervari et al [14] reported that a functional variant in the 3`-untranslated region (3`UTR) of PDYN, rs2235749, impairs the binding of miR-365 as well as PDYN expression. This finding may be a novel mechanism involving miR-365-PDYN interaction relevant to susceptibility to addiction. 

There are some limitations in the current study, one of which is the relative small sample sizes Second we did not perform stratification by sex due to small samples of females in the groups. Third, we evaluated 4 variants of the PDYN gene. Other genetic variants of these genes should also be evaluated. There is no clear explanation for departure from HWE regarding rs910080 and rs2235749 variants. It may be due to genetic drift. 

In summary, our findings support an association between *PDYN* rs910080 variant and risk of heroin addiction in a sample of Iranian population. Further investigation with larger sample sizes and diverse ethnicities are required to authenticate our findings.
